# Toward Meaningful Cultural Adaptation Across Implementation Stages: Lessons Learned From a Culturally Based HIV Stigma Intervention in Gaborone, Botswana

**DOI:** 10.9745/GHSP-D-22-00232

**Published:** 2022-12-21

**Authors:** Ohemaa B. Poku, Evan L. Eschliman, Rayna Y. Wang, Shathani Rampa, Haitisha Mehta, Patlo Entaile, Tingyu Li, Valerie W. Jackson, Ari Ho-Foster, Michael B. Blank, Lawrence H. Yang

**Affiliations:** a HIV Center for Clinical and Behavioral Studies, Division of Gender, Sexuality, and Health, Columbia University and New York State Psychiatric Institute, New York, NY, USA.; b Department of Health, Behavior and Society, Johns Hopkins Bloomberg School of Public Health, Baltimore, MD, USA.; c New York Coalition for Asian American Mental Health, New York, NY, USA.; d Department of Psychology, Queens College, City University of New York, Queens, NY, USA.; e Department of Counseling and Clinical Psychology, Teachers College, Columbia University, New York, NY, USA.; f Botswana-UPenn Partnership, Gaborone, Botswana.; g Department of Counseling and Clinical Psychology, Teachers College, Columbia University, New York, NY, USA.; h Department of Anesthesiology, University of California San Francisco, San Francisco, CA, USA.; i Research and Graduate Studies, Faculty of Medicine, University of Botswana, Gaborone, Botswana; Division of Infectious Disease, Department of Medicine, Perelman School of Medicine, University of Pennsylvania, Philadelphia, PA, USA.; j Perelman School of Medicine, University of Pennsylvania, Philadelphia, PA, USA.; k Department of Social and Behavioral Sciences, School of Global Public Health, New York University, New York, NY, USA; Department of Epidemiology, Mailman School of Public Health, Columbia University, New York, NY, USA.

## Abstract

A culturally based stigma intervention for pregnant women living with HIV in Gaborone, Botswana highlights the importance of conceptualizing and formalizing cultural adaptation across all stages of implementation.

## Key Messages

- We share lessons learned on how each stage of the implementation process of a culturally based stigma intervention benefited from cultural adaptation—including incorporating interexchange of knowledge during formative work, leveraging bilingualism and the cultural familiarity of local collaborators during the intervention, and prioritizing the concerns of local collaborators to inform intervention sustainment and scale-up.
- The implementation process could have been improved by more fully formalizing and recognizing the steps taken by local collaborators to culturally adapt throughout design and implementation.
- Lessons learned from this demonstration case can inform scale-up and sustainment of interventions whose implementation relies—either explicitly or implicitly—on cultural adaptation and local collaborators.
- Programs can benefit from building relationships of mutual respect and reciprocity with local collaborators and formally recognizing and supporting their contributions to cultural adaptation throughout the stages of implementation.

## INTRODUCTION

Being responsive to cultural, linguistic, and socioeconomic differences between intervention teams and participants is crucial for prudent, responsible, and effective implementation of global health interventions. A variety of concepts, approaches, and models have been proposed to ensure that the intervention resonates with its priority population, such as cultural competency,^1^^–^^4^ structural competency,^5^ cultural humility,^6^ trauma-informed approaches,^7^^–^^9^ and the biopsychosocial model.^10^ Additionally, methodologies such as community-based participatory research and other community-engaged methods also seek to coproduce research and interventions with their priority populations.^11^^–^^15^ These concepts, approaches, models, and methods vary in their emphases on interpersonal interactions, multilevel structures and systems, definitions of skills or processes, and redistribution of power. However, they have a shared focus on critically examining how various factors influence the care provided to individuals or groups, and they share a common goal to improve service provision and ultimately reduce disparities in care.

Implementation science also values fit between the intervention and the priority population. One way to help achieve fit that has been discussed in the context of implementation science is cultural adaptation, defined by Bernal et al.^16^ as “systematic modification of an EBT [evidence-based treatment] or intervention protocol to consider language, culture, and context in such a way that it is compatible with the client’s cultural patterns, meanings, and values.”^17^^,^^18^ Cabassa and Baumann^17^ argue that by integrating implementation science and cultural adaptation in mental health treatments, cultural factors can be considered explicitly at multiple levels. This, in turn, leads to clearer understandings of the context around an EBT that enhances practice and implementation outcomes and can also help navigation of the tension between adapting an intervention and maintaining fidelity to the EBT.^19^^–^^23^ Additionally, a growing body of literature and reviews show that cultural adaptation can be valuable in improving health outcomes^24^^,^^25^ and has the potential to improve intervention engagement and sustainability.^26^

A growing body of literature shows that cultural adaptation can be valuable in improving health outcomes and has the potential to improve intervention engagement and sustainability.

However, more exploration of the mechanisms by which cultural adaptation can and should happen is needed.^27^ The numerous models^24^^,^^27^^–^^30^ for cultural adaptation of health interventions that have been described and proposed share themes of gaining an understanding of the local context, considering the appropriateness of elements in the intervention, and adapting and implementing the intervention. In these models, however, strategies of engaging members of the local community and reflecting upon existing power structures are not consistently described. These considerations may be included during needs assessments, but they are not always reported in later stages of interventions.

Moreover, the terms used in this field and this article, such as culture, cultural adaptation, and EBTs have their own limitations. These terms have been conceptualized in ways that can perpetuate systems of oppression and marginalization. For example, the term culture has historically been used in the United States mostly in reference to minoritized racial and ethnic groups, a practice that can perpetuate an oppressive tradition in which “culture becomes a source of deviance…and Euro-American culture becomes the de facto norm and ‘standard.’”^31^ Health-related behaviors of participants have accordingly been too frequently and inappropriately framed as “cultural” when participants are also dynamically impacted by social, economic, and political environments.^31^ Furthermore, although EBTs have been grounded in rigorous testing processes, EBT research can also be inequitable as they have been historically tested with and published by individuals who work or study in academic settings.^32^ In sum, cultural adaptations need to occur in response not only to White, Eurocentric conceptualizations of culture but also to broader intersecting sociohistorical, structural, and contextual factors that impact research teams and participants.

In this commentary, we add to the burgeoning literature on the intersection of cultural adaptation frameworks and implementation science—while remaining cognizant of their limitations—by presenting the demonstration case of a culturally based stigma intervention for pregnant women living with HIV in Botswana called Mothers Moving towards Empowerment (MME; pronounced “mm-eh,” a Setswana term for a respected woman).

A primary emphasis in this case is the critical role of local collaborators. We consider a local collaborator to be anyone from the same cultural context as the intervention’s participants who also partners with or is a part of the research team. Local collaborators can include data collectors, study coordinators, and intervention facilitators and peer coleaders—with these roles not being mutually exclusive. Although participants in our intervention were also integral to intervention implementation, we consider them separately from local collaborators in this article because they were recruited and consented to be enrolled in the intervention and therefore were not a formalized part of the research team.

We use the stages of the Exploration, Preparation, Implementation, Sustainment (EPIS) framework^33^ to serve as an organizational framework to discuss cultural adaptation across all stages of implementation. The Table provides a high-level summary of the steps taken toward cultural adaptation and key findings from each stage that informed the implementation of MME, as well as suggestions to recognize the involvement and roles of local collaborators more formally across each EPIS stage.

**TABLE.** Cultural Adaptation Related to MME Throughout EPIS Stages and Suggestions for Further Formalizing the Involvement of Local Collaborators

**EPIS Stage**	**Examples of Cultural Adaptation Steps Taken**	**Relevant Key Findings**	**Ways to More Fully Formalize Involvement and Roles of Local Collaborators**
Exploration	Local collaborators conducted formative interviews and focus group discussions with community members. Key informants in the community reviewed and contextualized the findings. Local stakeholders, content experts, and potential implementers in community formed a steering committee.	Formative qualitative research revealed that HIV-related stigma in the local context is heavily gendered.	Identify and respond to power structures within research team and between research team and research participants (e.g., cultural, linguistic, and socioeconomic differences). Identify and respond to power structures relevant to the intervention and work planned (e.g., gender), which may also influence the identification, recruitment, and engagement of local collaborators. Actively involve local collaborators in formative cultural adaptation work. Be equipped with adequate financial and infrastructural resources for recruitment, training, and payment of local collaborators.
Preparation	Steering committee (including local clinical experts, psychology graduate students, and peer coleaders) met to develop and adapt manual. Binational training between local collaborators and U.S.-based clinicians.	Women register late for antenatal care, so intervention recruitment strategies were changed accordingly to meet women when they typically visit clinics. “Being free” was identified as a local idiom reflecting liberation and empowerment, so the cognitive behavioral therapy module was renamed “The Road to Self-Acceptance and Freedom.” Feedback from local collaborators led to the development of a graduation ceremony component involving the bestowing of ceremonial shawls.	Actively seek out discussions of potential barriers and facilitators of intervention with local collaborators and other local key informants, even if they are not “researchers” in the formal sense. Consider, identify, and respond to power imbalances at play during binational training. Readily incorporate local views, concepts, and idioms into curriculum. Note these contributions for evaluation in follow-up to monitor resonance with participants.
Intervention	Local collaborators engaged in their own fidelity checks (in addition to formalized fidelity checks outlined in the study protocol with a clinical psychologist) and made real-time changes to curriculum (e.g., took notes of what worked best and what needed modification after each session, including terms and language use) based on participants’ feedback. Local collaborators took the time to listen to participants and relay experiences to the research team.	Local collaborators changed timing of intervention based on clinic and participant availability and schedule. Local collaborators were able to engage participants by flexibly switching between languages during the intervention.	Give local collaborators resources and support to listen to participant feedback and make the changes (e.g., in format, content) they see fit. Engage and retain local collaborators who are bilingual when possible or appropriate. Include language and other cultural considerations in agendas of fidelity checks, training sessions, or other meetings with local collaborators. Consider and identify measures, outcomes, and data reporting methods that resonate with local collaborators and communities.
Sustainment	Local collaborators shared challenges faced during implementation.	A lack of translation of the MME manual into Setswana may hinder implementation in settings outside of Gaborone. The obligation to work extra hours to host evening sessions may disincentivize implementation at other clinics. Clinic staff were involved in antenatal care phase for participants but would not be involved in women’s experiences postpartum, which impacts sustainment of the MME intervention and continuity of nonstigmatizing care.	Prioritize listening to concerns and feedback from local collaborators and participants to inform sustainment. Start seeking and noting feedback from local collaborators and participants relevant to sustainment as far back as the exploration stage. Consider, budget for, and provide adequate financial and infrastructural resources for retention of local collaborators as well as recruitment and training of additional local collaborators. Support local collaborators in pursuing self-identified professional goals as research projects expand.

Abbreviations: EPIS, Exploration, Preparation, Implementation, Sustainment; MME, Mothers Moving towards Empowerment.

## EXPLORATION AND PREPARATION

Exploration is the EPIS stage that entails identifying emergent or existing health needs within the community, followed by consideration of the best evidence-based approach to meet those needs.^33^ The research group and/or local stakeholder(s) explore ways to address the identified need by adapting the intervention to operate at or within system, organization, and individual level(s). This initial inquiry is followed by preparation, the second phase of the EPIS framework, in which the primary objectives are to identify barriers and facilitators to implementation, recognize resources and supports required to necessitate adaptation, and develop the specifics of the intervention.^33^ Incorporation of local cultural meanings can be and is often done during these stages but sometimes is not formalized or recognized as cultural adaptation. Further, researchers can be untrustworthy to local participants, especially in initial stages of research, meaning local cultural meanings may be incorrectly extracted, misused, or appropriated by researchers when not accounting for existing power dynamics. It is essential that research teams build relationships with local collaborators with mutual respect and reciprocity, which requires sharing power.^15^

Our formative qualitative research was motivated by the identification of HIV-related stigma as a major barrier to taking antiretroviral therapy among women in Botswana, particularly postpartum. Local collaborators recommended to our team by other local collaborators—in this case, study and project managers who were already affiliated with international university partnerships—were instrumental in conducting in-depth interviews and focus group discussions with people living with HIV and people whose HIV status was not asked about. Some of these new local collaborators later became key members of the research team throughout the subsequent stages described in later sections.

After data collection, the What Matters Most theory^34^^,^^35^ was then used to identify aspects of life that “matter most” and allow a person to achieve “full personhood” within their specific local context (i.e., contemporary Setswana culture). Results revealed that HIV-related stigma is heavily gendered, “being a respected mother” helps women achieve “full womanhood” and resist stigma, and HIV-related stigma is felt most acutely by women when living with HIV threatens the status of “being a respected mother.”^36^ Identifying the salience of gender and gender norms during this early phase guided many of the subsequent steps involved in the cultural adaptation. These results also underwent review by key informants from the community and allowed for further contextualization of the findings.^37^

Then, to facilitate further clarification and implementation of these core concepts that were identified, the investigating team collaborated with local stakeholders (e.g., the Greater Gaborone District Health Management Team) and those with expertise working with women in Botswana (i.e., an HIV care pediatrician, psychiatrist, and epidemiologist) to form a steering committee. This steering committee guided the cultural adaptation of a previously implemented^38^ evidence-based stigma intervention. The resulting intervention, MME, consists of group sessions led by women living with HIV and organized around the components of psychoeducation for HIV, challenging inaccurate stereotypes of HIV, and identifying behavioral coping responses for HIV-related discrimination.^39^

For MME, preparation also included committee members convening weekly (on Skype and in person) to develop the MME manual and ensure its responsiveness to the cultural context. A local clinical expert, psychology graduates with training in counseling, and peer coleaders (i.e., women who identified as living with HIV during preg-nancy in the past) were engaged in developing the manual. The peer coleaders, also referred to in this article as local collaborators, were later trained on the intervention delivery skills through a partnership between local and U.S.-based clinicians. Discussions with the peer coleaders and local clinical experts surrounding potential barriers and enablers of MME also enhanced the ability of the entire research team to address these barriers while working toward a partnership with and leveraging the skills of the facilitators before implementation. This intensive, collaborative step made it clear that both communication with local collaborators and adaptation of the intervention materials need to be iterative and ongoing. For example, the team learned from local collaborators that women commonly register for antenatal care later in pregnancy than the recruitment criteria had originally planned for. This led to MME focusing recruitment efforts on women later in pregnancy when mothers typically visit antenatal clinics, which in turn led to more efficient outreach for study recruitment and sharing information regarding the vertical transmission of HIV.

Discussions with peer coleaders and local clinical experts over potential barriers and enablers enhanced the ability of the entire research team to address these obstacles before implementation.

Additionally, cultural components were added to the intervention via the incorporation of local idioms. For example, “being free” was identified as a local idiom reflecting a sort of internal liberation in which an individual feels empowered and as having no bondages—that “nothing’s holding me back.” Accordingly, the cognitive behavioral therapy intervention module was renamed “The Road to Self-Acceptance and Freedom.” Feedback from local collaborators also led to the develop-ment of a graduation ceremony component involving the bestowing of ceremonial shawls similar to those often bestowed at marriage that signal maturity and womanhood. This provided another way to convey to participants that they can indeed achieve "what matters most" (i.e., “being a respected mother”) while living with HIV. Later, feedback from participants suggested this ceremonial aspect was indeed an empowering tool for mothers that enabled reclamation of freedom and agency, as well as the possibility of being a woman respected in her community.

## IMPLEMENTATION

Next, during the implementation phase, an adapted intervention is initiated into its intended systems and organizations.^33^ Ongoing efforts to monitor the implementation process while adjusting implementing strategies are crucial for translating research into effective practice and enhanced through a cadre of local collaborators who provide iterative feedback. The EPIS framework delineates several outer and inner contextual factors that may potentially influence implementation effectiveness.^33^^,^^40^ In the framework, the outer context refers to the sociopolitical environment outside the organization such as funding resources, service contracting, and leadership for implementation. The inner context refers to organizational characteristics such as organizational structures, priorities and goals, culture and climate, and readiness to implement the intervention. Together, the outer and inner components reflect the dynamic and multilayered nature of the implementation process, but little in the EPIS framework explicitly names how these outer and inner contextual factors relate to cultural adaptation.

In the case of MME, it was largely local collaborators who drove cultural adaptation during implementation. When hearing reflections from local collaborators, it became evident that in many ways, they were undertaking their own type of fidelity checks when facilitating each session, taking note of what worked best and what needed modification. It should be noted that per our study’s protocol, fidelity checks with a clinical psychologist were a core component of the intervention—not only to ensure delivery of the intervention but also to provide space for facilitators to reflect and receive support. In turn, as they facilitated the intervention, local collaborators then seamlessly and in real time made these changes at the microlevel that are now informing the overall macrolevel of the intervention itself in ways necessary for scale-up to occur. For example, local collaborators worked closely with participants and the clinics where the intervention was sited to minimize burden and inconvenience for all parties involved (e.g., peer coleaders and study coordinators asked the clinic to change the intervention time to ensure participants had safe transportation arranged after each session was complete). This identification of what tailoring is necessary to meet the needs of both participants and those implementing the intervention, especially for the sake of fidelity, is known to be an integral step in comprehensively applying an intervention to a specific setting.^41^

Local collaborators largely drove cultural adaptation during implementation, taking note of what worked best and what needed modifications while facilitating sessions.

Another specific insight in this vein shared by local collaborators is that a “choice” between languages may not be wholly binary. Although English is the official language in Botswana, Setswana is the national and most widely spoken language. Local collaborators shared their observations that being open to using both English and Setswana allowed participants to express themselves openly and confidently. This iterative process of flexibly switching between the 2 languages was core to the success of MME; 1 poignant statement shared by a local collaborator was that “the second you show yourself to lack proficiency in a language, it creates a barrier.”

Local collaborators also shared that in some cases, participants even took on the role of a facilitator to help with translation concerns and help each other convey their ideas more accurately. It was clear that switching between languages happens naturally during real-time conversation and that challenges arise after the fact to find the “perfect term” to capture discussions. Consideration of the spectrum of bilingualism and how to enable MME’s responsiveness to it clearly helped enable its success, as a similar sensitivity could for other interventions. Additionally, when bilingual facilitators from the local culture adapt the intervention based on linguistic norms of the community, it also redistributes power by giving local collab-orators a degree of control over intervention content that was largely developed by nonlocal, English-speaking researchers.

Feedback provided by local collaborators indicated that their ability to adapt and their willingness to innovate led to a depth and breadth of resourcefulness that enhanced implementation of MME and, ultimately, made the intervention work better for participants. For example, the local collaborators in these roles took the time to listen and accurately capture experiences, which made the participants feel valued and that they were equals. This also afforded a strong relationship between local collaborators and the participants that kept them engaged throughout the intervention as well as aided in the retention rates per session. Additional outcomes and successes of MME, such as the decreases in HIV-related stigma and depressive symptoms, are published separately.^42^ How outcomes and “success” are measured, reported, and distributed also deserves reflection and attention. Although discussion around cultural adaptation of evaluation lies outside the scope of our article, it was clear that the adaptations made by local collaborators in the process of implementation should be celebrated and recognized in evaluation efforts. A greater understanding of the many unspoken but essential roles local collaborators had in facilitating MME enhances not only the future fidelity and suitability of the intervention, but also lends further understanding of the ways these critical personnel could be recognized and supported. Engaging with collaborators and fully recognizing the ways they adapt the actual delivery of an intervention has been noted as a fundamental step in intervention implementation,^41^ and it is particularly necessary for cultural adaptation as well.

## SUSTAINMENT

Sustainment, the last phase in the EPIS framework, is viewed as “the continued use of an innovation in practice”^33^ and plays a role similar to other phases that have been denoted in the literature such as “maintenance”^43^ or “self-regulation.”^44^ It is also important to note that definitions of sustainability in implementation science remain highly varied between studies and implementation frameworks.^45^^,^^46^ In this article, however, we consider sustainment as a phase that follows the first cycle of use of an intervention or practice (i.e., a pilot).

In the case of MME, our sustainment phase includes considerations for scaling up the intervention for potential national application, which may not be the case for other interventions applying the EPIS framework. Sustainment—and relatedly, sustainability—are increasingly being acknowledged as key areas of improvement for the implementation of culturally adapted and other interventions.^26^^,^^47^ Leading scholars in the field recognize the need for much more research and engagement around these concepts to work toward a beneficial understanding of sustainability and how to best ensure it,^48^ and very little to date has been researched or written about the role of cultural adaptation in the sustainment phase of interventions.

Through conducting interviews with local collaborators involved in MME, it became clear that these study team members also had insights and experiences related to sustainment and sustainability. For example, local collaborators explained that language-related difficulties will likely arise when scaling up MME. Specifically, local collab-orators pointed out it will be necessary to have a Setswana version of the intervention manual alongside the existing Setswana versions of participant handouts. While real-time flexibility in language use and other linguistic adaptations were a key strength of the facilitators during this intervention, local collaborators felt that having both English and Setswana versions of the manual to see suggested translations of concepts would reduce the burden for session leaders in the future. Relying solely on the creation of real-time translations could also reduce fidelity of the intervention by allowing for greater variation in how concepts integral to the intervention (e.g., stigma, motherhood) are introduced to the groups. In addition, these linguistic adaptations may need to change at different intervention sites because key vocabulary may not overlap in regions outside of Gaborone, and other dialects and languages would need to be incorporated.

Local collaborators shared insights related to sustainment, such as language-related difficulties that will likely arise when scaling up MME.

The local collaborators also raised human resources concerns relevant to sustainment and sustainability. It was noted that group sessions often took place after clinic staff had left work for the day. This meant that any clinical staff who attended the group sessions had to stay after work, and if clinical staff did not want to stay, it was also more difficult to obtain answers to women’s clinical questions around HIV that may have arisen during the group session. Similarly, local collaborators expressed concerns that these clinical staff worked only in antenatal care, and therefore would not necessarily be in participants’ lives postpartum. In considering scale-up, this information is invaluable to accurately determine the timing and resources needed to successfully implement MME in a way that does not overly strain the clinics at which it will be sited and best ensures that participants have greater continuity in nonstigmatizing care.

As we plan to scale up MME, we recognize the important role that local collaborators can play in cultural adaptation broadly, and in turn, how they can contribute greatly to scale-up and sustainability. A critical factor in any next steps to sustain or scale up MME will be the identification and recruitment of additional local collaborators who have the same dedication and care that has undoubtedly contributed to MME’s success. Additionally, local collaborators should be given the resources and support to pursue their self-identified professional goals when possible as interventions and their teams expand. Few published research efforts to promote sustainability proactively include, or at least formalize, the experiences, expertise, and insights that local collaborators can provide. More often, efforts are focused on capturing opinions of community members and local experts who have not been directly involved in the intervention, thereby potentially excluding the local collaborators—and participants—who may have important insights about what will and will not work. Looking at the influence that local collaborators can and do have on sustainment can reveal beneficial synergies in common sustainment strategies such as ensuring and measuring cultural fit,^26^ delineating implementation strategies (e.g., through “mapping”), and developing strategic action plans.^47^^–^^49^ It is possible that study teams do obtain regular feedback from local collaborators via qualitative or informal methods; however, the lack of reporting of these processes means each study team may be using their own ad hoc methods with little opportunity to build best practice or strategies around interfacing with local collaborators around sustainment, sustainability, and scale-up.

## CONCLUSION

By situating the experience of the cultural adaptation process that resulted in the development of MME within the EPIS framework, this article sought to capture the ways in which the implementation process benefited from cultural adaptation—especially via the efforts of community members, local collaborators, and participants. At the same time, this process could have been improved by more fully formalizing and recognizing the steps taken to culturally adapt throughout the implementation, especially uplifting the critical role of local collaborators; suggestions to this end are provided in the Table.

Implementation science would also benefit from additional research on the roles of local collaborators in cultural adaptation. Clearly, concepts within community-engaged research, such as the exchange of knowledge with community providers, building relationships with mutual respect and reciprocity, commitment to ongoing communication, and leadership development among research teams^14^^,^^50^^–^^52^ are relevant to creating a more equitable research and implementation process. Additionally, actions toward the goal of decolonizing global health require approaches and practices that acknowledge the history of colonialism and actively resist the perpetuation of patterns of oppression, exclusion, and exploitation.^53^^,^^54^ As more equitable strategies and practices emerge, it is also important to recognize that strategies may—and should—vary for each specific context and project given the complex power dynamics at play. The lessons learned from this demonstration case can serve to inform scale-up and sustainment of not only this specific intervention, MME, but also other interventions whose implementation relies—either explicitly or implicitly—on cultural adaptation and local collaborators.

## Notes

**Peer Reviewed**

**First published online:** November 22, 2022.
